# Filamin repeat segments required for photosensory signalling in *Dictyostelium discoideum*

**DOI:** 10.1186/1471-2121-8-48

**Published:** 2007-11-12

**Authors:** Sarah J Annesley, Esther Bandala-Sanchez, Afsar U Ahmed, Paul R Fisher

**Affiliations:** 1 Department of Microbiology, La Trobe University, Vic., Australia

## Abstract

**Background:**

Filamin is an actin binding protein which is ubiquitous in eukaryotes and its basic structure is well conserved – an N-terminal actin binding domain followed by a series of repeated segments which vary in number in different organisms. *D. discoideum *is a well established model organism for the study of signalling pathways and the actin cytoskeleton and as such makes an excellent organism in which to study filamin. Ddfilamin plays a putative role as a scaffolding protein in a photosensory signalling pathway and this role is thought to be mediated by the unusual repeat segments in the rod domain.

**Results:**

To study the role of filamin in phototaxis, a filamin null mutant, HG1264, was transformed with constructs each of which expressed wild type filamin or a mutant filamin with a deletion of one of the repeat segments. Transformants expressing the full length filamin to wild type levels completely rescued the phototaxis defect in HG1264, however if filamin was expressed at lower than wild type levels the phototaxis defect was not restored. The transformants lacking any one of the repeat segments 2–6 retained defective phototaxis and thermotaxis phenotypes, whereas transformants expressing filaminΔ1 exhibited a range of partial complementation of the phototaxis phenotype which was related to expression levels. Immunofluorescence microscopy showed that filamin lacking any of the repeat segments still localised to the same actin rich areas as wild type filamin. Ddfilamin interacts with RasD and IP experiments demonstrated that this interaction did not rely upon any single repeat segment or the actin binding domain.

**Conclusion:**

This paper demonstrates that wild type levels of filamin expression are essential for the formation of functional photosensory signalling complexes and that each of the repeat segments 2–6 are essential for filamins role in phototaxis. By contrast, repeat segment 1 is not essential provided the mutated filamin lacking repeat segment 1 is expressed at a high enough level. The defects in photo/thermosensory signal transduction caused by the absence of the repeats are due neither to mislocalisation of filamin nor to the loss of RasD recruitment to the previously described photosensory signalling complex.

## Background

The actin cytoskeleton in eukaryotic cells is an essential structure for a wide variety of functions including muscle contraction, cell movement, cytokinesis, cytoplasmic organisation and intracellular transport [1]. The actin cytoskeleton is highly dynamic and can be reorganised within seconds after chemotactic stimulation and these changes are regulated by a large number of actin binding proteins. Filamin is an actin binding protein which can crosslink actin filaments helping to stabilise the 3D cortical actin network. The basic architecture of filamin is well conserved and consists of an actin binding domain at the N-terminus followed by a C-terminal rod domain comprised of numerous repeat segments ranging from 4 in *C. elegans *to 24 in mammalian cells. Each repeat in the rod domain consists of approximately 100 residues and forms an immunoglobulin like fold [2]. Such immunoglobulin folds have been found in various proteins and are responsible for protein-protein interactions [3]. Mammalian filamin (ABP280) has been reported to interact directly with at least 30 proteins [4] including transmembrane receptors, second messenger-associated proteins, protein kinases, phosphatases and cytoskeletal proteins and these interactions have been shown to require one or more of the repeat elements in the rod domain. By binding various members of a signalling complex filamin is thought to act as the scaffold holding many of the components in a complex in the correct location for efficient transduction of the signal.

*D. discoideum *is an ideal model eukaryote for the study of the actin cytoskeleton and signal transduction pathways. Many actin binding proteins and cytoskeletal components have been identified in *Dictyostelium *and all of these elements are also found in mammalian cells [5]. *D. discoideum *is amenable to many cell biological and molecular genetic techniques and its haploid nature aids in the isolation of mutants. The life cycle of *D. discoideum *is complex with unicellular and multicellular stages of development. Upon starvation the amoebae secrete cAMP which makes them attractive to each other and initiates the aggregation of cells to form a multicellular organism, the slug. The slug is phototactic and thermotactic which allows the slug to migrate to an optimal location for the formation of the fruiting body and eventual release of the spores. It has been estimated that 20–55 proteins may be involved in slug behaviour [6]. Some of the signalling molecules identified to date include second messengers such as cAMP [7], cGMP [6], IP_3 _[8] and Ca^2+ ^[9], as well as the GTPase RasD [10] and the guanine nucleotide exchange factors GefE and GefL [11]. The proteins required for normal photosensory signal transduction also include some actin-binding proteins such as GRP125 [12], villidin [13], Cyclase Associated Protein (CAP) [14] and filamin [15], but a variety of other actin-binding proteins are not needed for normal phototaxis [16].

Several independent filamin null mutants have been created and all displayed a severe defect in slug behaviour with filamin mutant slugs showing deranged phototactic and thermotactic responses [16]. The rod domain of Ddfilamin contains six repeat segments and has recently been shown to bind FIP (filamin interacting protein) a protein of unknown function [17]. This interaction required the rod domain. Here we show using complementation studies that repeats 2–6 in the rod domain are each essential for slug phototaxis while repeat 1 is not required if expression levels are sufficiently high. Immunofluorescence studies also show that none of the repeat segments are involved in the localisation of filamin.

Filamin has been shown to form a putative photosensory signalling complex with RasD, ErkB, GRP125 and PKB [18]. Of these proteins RasD [10] and GRP125 [12] have been shown to be essential for photosensory responses in *D. discoideum*. Here we show that RasD binding to the complex does not depend on any single repeat element in the rod domain.

## Results

### Complementation of the phototaxis phenotype depends upon the level of wild type filamin expression

**Figure 1:**
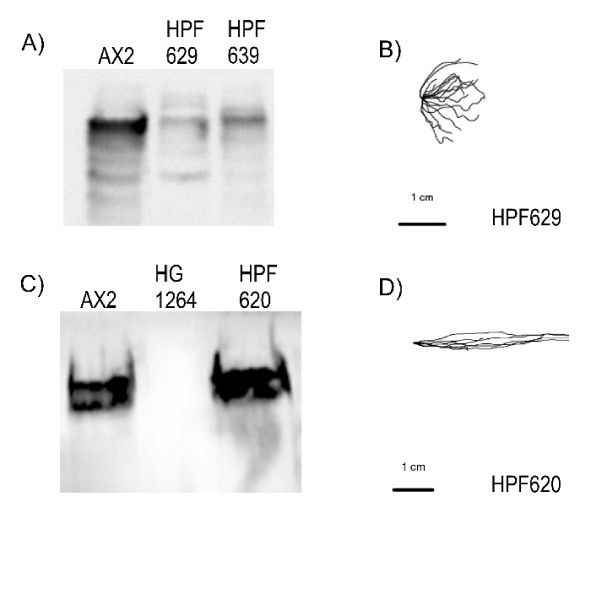
**Complementation of filamin null mutant HG1264 depends upon the level of filamin expression**. (A) Western blot of proteins from AX2 and two filamin mutant transformants, HPF629 and HPF639 ectopically expressing filamin. The two transformants expressed filamin at a lower level than that observed in the wild type. (B) The slug trails of HPF629 were digitised and this transformant displayed a defective phototaxis phenotype as observed in the filamin mutant from which this strain was derived, HG1264. The light source is to the right of the figure. (C) Western blot using the antifilamin antibody against total cellular protein from AX2 (lane 1, wild type), HG1264 (lane 2, filamin null mutant) and HPF620 (lane 3, filamin mutant ectopically expressing filamin). No filamin can be detected in the filamin mutant whereas filamin is expressed in HPF620 to a level comparable to that of the wild type. (D) The slug trails of HPF620 were digitised and this transformant displayed a wild type phototaxis phenotype. The light source is to the right of the figure.

The filamin null mutant HG1264 displays a severely defective slug phototactic and thermotactic response [15,16]. In an attempt to rescue this defect, we transformed HG1264 with constructs which expressed full length filamin. We found in qualitative tests that the phototaxis phenotype varied amongst the transformants, with some (e.g. HPF629, HPF639) exhibiting severely deranged phototaxis whilst most exhibited wild type phototaxis (e.g. HPF620). A Western blot revealed that these transformants expressed filamin at different levels. HPF629 and HPF639, both of which exhibited a deranged phototactic phenotype, were found to express filamin at levels well below that of wild type (Figure 1A, B). By contrast, HPF620, whose phototactic phenotype had been restored, expressed filamin at wild type if not higher levels (Figure 1C, D). This suggests that the wild type level of filamin expression is essential for efficient phototactic signalling.

### All but the first of the filamin repeat segments are required for phototaxis and thermotaxis

The structure of filamin consists of an N-terminal actin binding domain, which is highly conserved amongst many actin binding proteins and a C-terminal rod domain constructed of six repeat segments that are conserved amongst filamin proteins from different organisms but not found in other actin-binding proteins. As the repeat segments are the defining feature of filamin it was suggested previously that the protein's role in phototaxis might depend on its rod domain and perhaps on particular repeats in the rod domain [16]. To test this hypothesis various constructs were created which contained internal deletions of one of the repeat segments and these were transformed into the filamin null mutant in an attempt to rescue the phototaxis defect.

**Figure 2:**
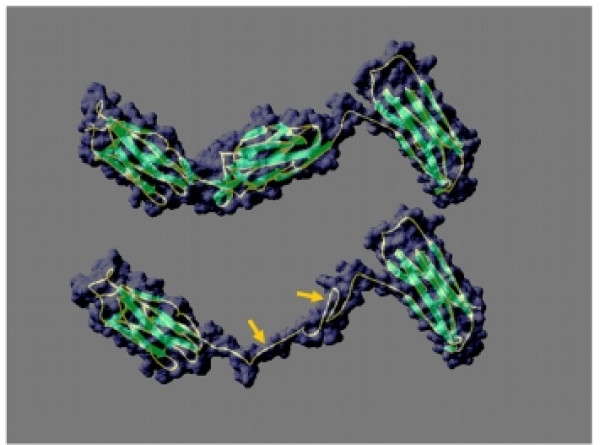
**Prediction of the structure of filaminΔ5**. Structural prediction of a region of filaminΔ5 corresponding to segments 4 through 6 of the full length filamin (amino acids 547 to 838, including the first two β strands of segment 5) but with residues 672 through 743 (most of segment 5) deleted (DDB0201554). The prediction was made using the SwissModel first approach method accessed through the ExPASy Proteomics Server [20-25]. The resulting prediction was based on known *D. discoideum *and human filamin structures. The structures for repeat segments 4–6 have been determined by Popowitcz *et al. *[19] and as the repeats are very similar it is believed that repeat segments 1–3 would contain a similar structure to repeats 4 and 5. Therefore this figure uses the deletion of repeat segment 5 as an example which could be extrapolated for all the other repeat segments. The top panel is the known structure [19] of repeat segments 4 through to 6, the bottom panel shows the predicted structure for the corresponding region of filaminΔ5. This figure illustrates that by deleting all but the first two β strands of a repeat segment, it is possible to correctly fold and maintain the correct distance between the repeat segments that flank the deleted region. The gold arrows indicate the position of the sequences corresponding to the first two β strands of segment 5 in the wild type filamin structure.

**Figure 3:**
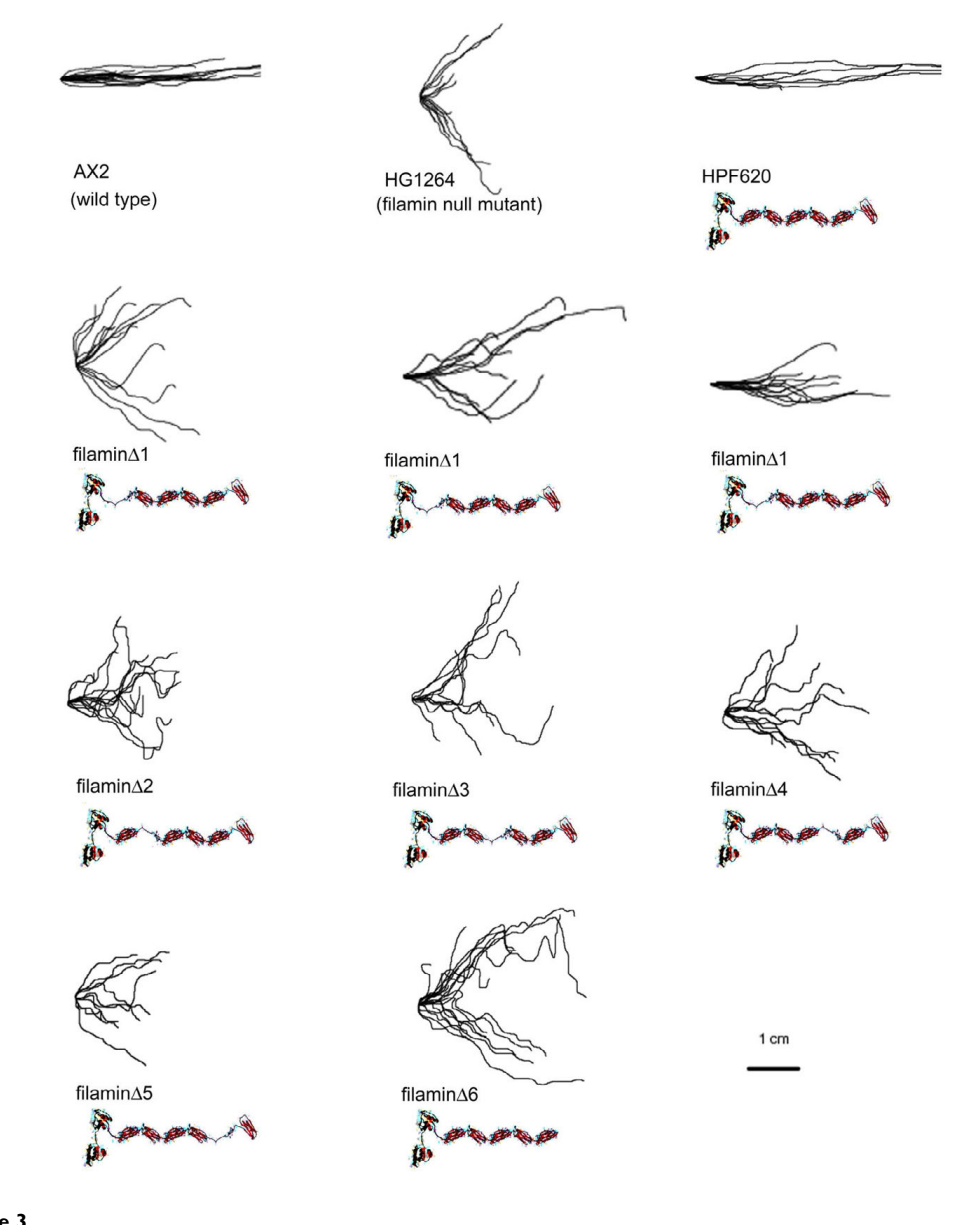
**Qualitative phototaxis by strains lacking repeat segments**. Digitised slug trails of wild type AX2, the filamin null mutant HG1264, the rescued strain HPF620 and the strains lacking one of the repeat segments. Slug trails were plotted from a common origin so that the source of light is to the right of the figures. Wild type slugs migrate directly towards the light source whereas filamin mutant slugs show disoriented, bimodal phototaxis with two preferred directions either side of the light source. The rescued strain HPF620 displays wild type phototaxis whereas the strains lacking repeat segments 2–6 display mutant phototaxis and transformants lacking the first repeat show phototaxis phenotypes ranging from wild type to mutant.

The filamin deletions (referred to as filaminΔ1 through filaminΔ6) were created by removing the amino acids encompassing the entire repeat with the exception of those encoding the first two β strands based on X-ray crystallography data by Popowicz *et al. *[19]. In each of the repeat segments, the first 2 β strands extend to the junction with the next repeat segment before the polypeptide backbone folds back upon itself to allow alignment of the next β strand. Inclusion of the first two β strands of the deleted repeat thus created a spacer that was predicted to maintain approximately the original separation between the preceding and following repeats. This could compensate for the absence of the deleted repeat, avoiding any structural or steric hindrance problems the lack of a repeat may pose. To test the feasibility of this idea, we modelled the structure of repeat segments 4 through 6 in filaminΔ5 using the first approach method at the Swiss Model Comparative Protein Modelling Server at the Swiss Institute of Bioinformatics [20] accessed through the ExPASy Proteomics Server [21-25]. Figure 2 shows the resulting structural prediction below the known X-ray crystal structure for *Dictyostelium *filamin segments 4 through 6. This prediction demonstrates that retaining the first two β strands may indeed provide a spacer of approximately the same length as a full length repeat segment. Approximately 100 transformants expressing a deletant filamin lacking one of repeat segments 2 through 6 were analysed and in no case was the wild type phototaxis phenotype restored. By contrast, a range of partial complementation of the phototaxis phenotype was observed in transformants expressing filaminΔ1 (Figure 3).

**Figure 4:**
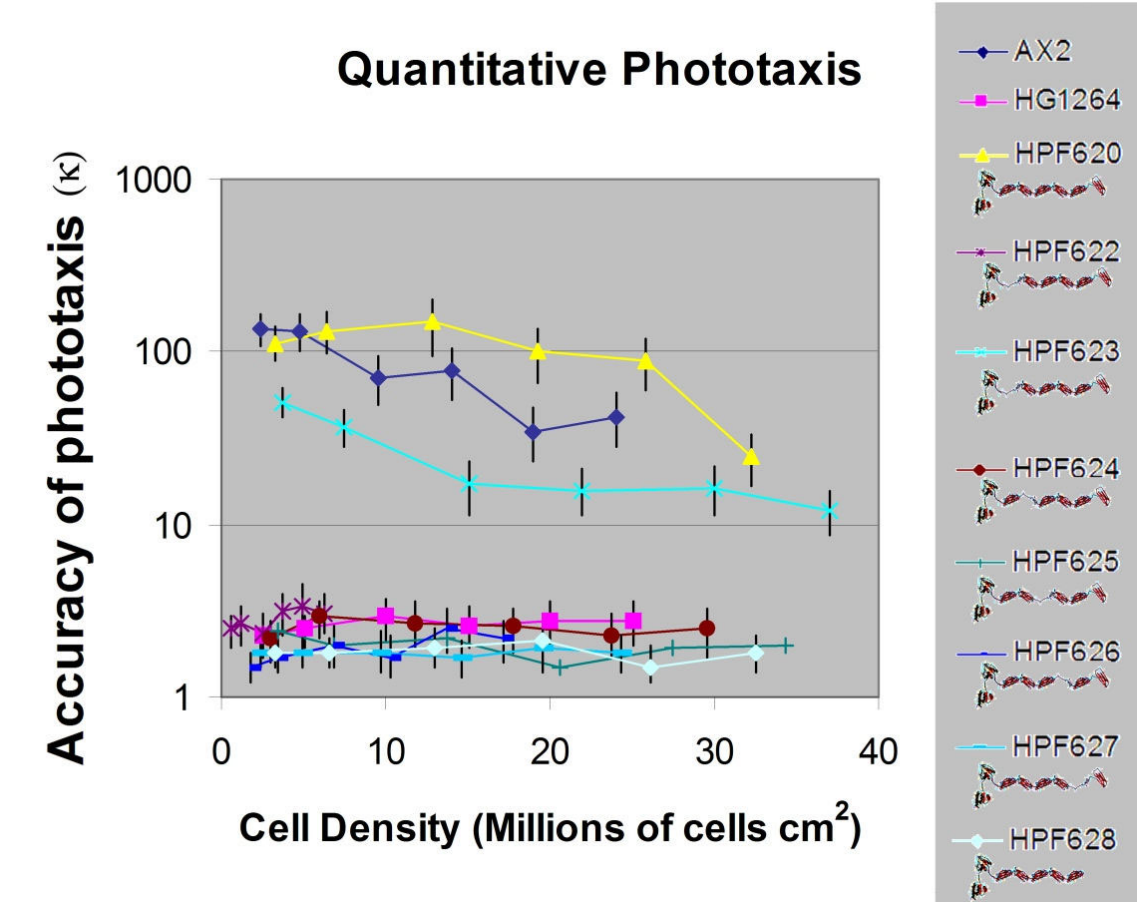
**Quantitative phototaxis of strains lacking repeat segments**. Phototaxis at defined cell densities was performed on wild type AX2, the filamin null mutant HG1264, the rescued strain HPF620 and the strains expressing filamin lacking one of the repeat segments as indicated by the cartoon under the strain name. The trails were digitised and analysed using directional statistics to determine their accuracies of phototaxis (κ) at the defined cell densities. Vertical bars represent 90% phototaxis than the wild type AX2, the original mutant (HG1264) displayed poor accuracies, as did all of the strains lacking repeat segments 2–6. Two transformants expressing filamin with a deletion of repeat one showed accuracies of phototaxis of approximately 1% (HPF622) or 50% (HPF623) of the wild type values.

To determine the extent of the complementation two strains expressing each of the mutant filamin forms were chosen for quantitative phototaxis (Figure 4). These experiments confirmed the differences amongst the strains. The transformant ectopically expressing full length filamin (HPF620) exhibited wild type accuracies of phototaxis at low densities and was, if anything, slightly less sensitive to the impairment of phototaxis that normally occurs at high cell densities [3]. The strains expressing filaminΔ2–6 showed no complementation of the phototaxis phenotype. Two strains expressing filaminΔ1 were chosen – one that in the qualitative tests largely restored the phenotype (HPF623) and one that did not (HPF622). The accuracy of phototaxis (κ) in the former was restored to approximately 50% that of wild type while in the latter no significant restoration of normal phototaxis was observed (κ ≅ 1% of wild type).

**Figure 5:**
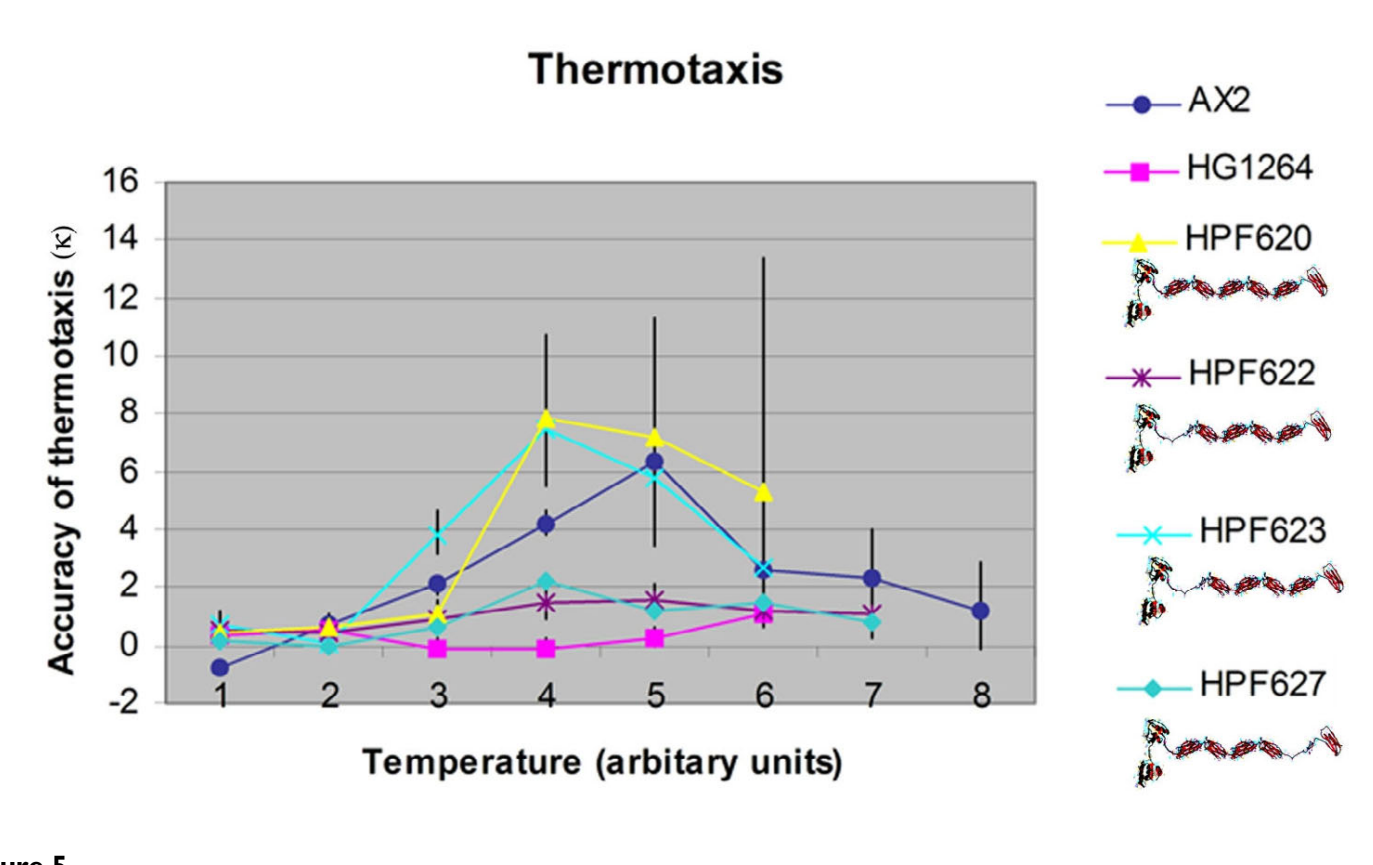
**Quantitative thermotaxis of strains lacking repeat segments**. Quantitative measurements of thermotaxis by slugs of wild type AX2, filamin null mutant HG1264, the rescued strain HPF620, two transformants expressing filaminΔ1 HPF622 and HPF623 and HPF627 a transformant expressing filaminΔ5. All transformants expressing filmainΔ2–6 showed similar thermotaxis phenotypes to HPF627 which was included as an example. Temperatures follow the previous convention of being designated 1 through 8 in arbitrary units. Separate calibration experiments have shown that these correspond to temperatures at the surface of the centre of each plate ranging from 14°C in 2°C steps to 28°C, with a temperature gradient at the agar surface of 0.2°C/cm. The slug trails were digitised and analysed using directional statistics [3]. Positive values indicate the accuracy of positive thermotaxis (κ) towards the warmth, negative values indicate the accuracy of negative thermotaxis (κ) towards the cold and a value of zero indicates there was no preferred direction. Vertical bars are 90% confidence limits. AX2, HPF620 and HPF623 all display high accuracies of positive thermotaxis at T4 and T5 and low positive accuracies of thermotaxis at the cooler and hotter temperatures with AX2 displaying negative accuracies of thermotaxis at T1. HG1264, HPF622 and HPF627 displayed small accuracies of thermotaxis at all temperature points. It is normal for the accuracy of thermotaxis by wild type AX2 slugs to decline above and below the growth temperature and either approach zero or even switch to negative thermotaxis at T1 [16, 39]. At some of the higher temperatures slugs of several of the strains did not migrate so thermotaxis could not be measured.

Quantitative thermotaxis experiments produced similar results (Figure 5). The transformants expressing filaminΔ2–6 showed no restoration of normal thermotaxis. The thermotaxis defect was completely rescued in HPF620 expressing full length filamin and in HPF623 expressing filaminΔ1, but little or no complementation was observed with the strain HPF622 (also expressing filaminΔ1).

**Figure 6:**
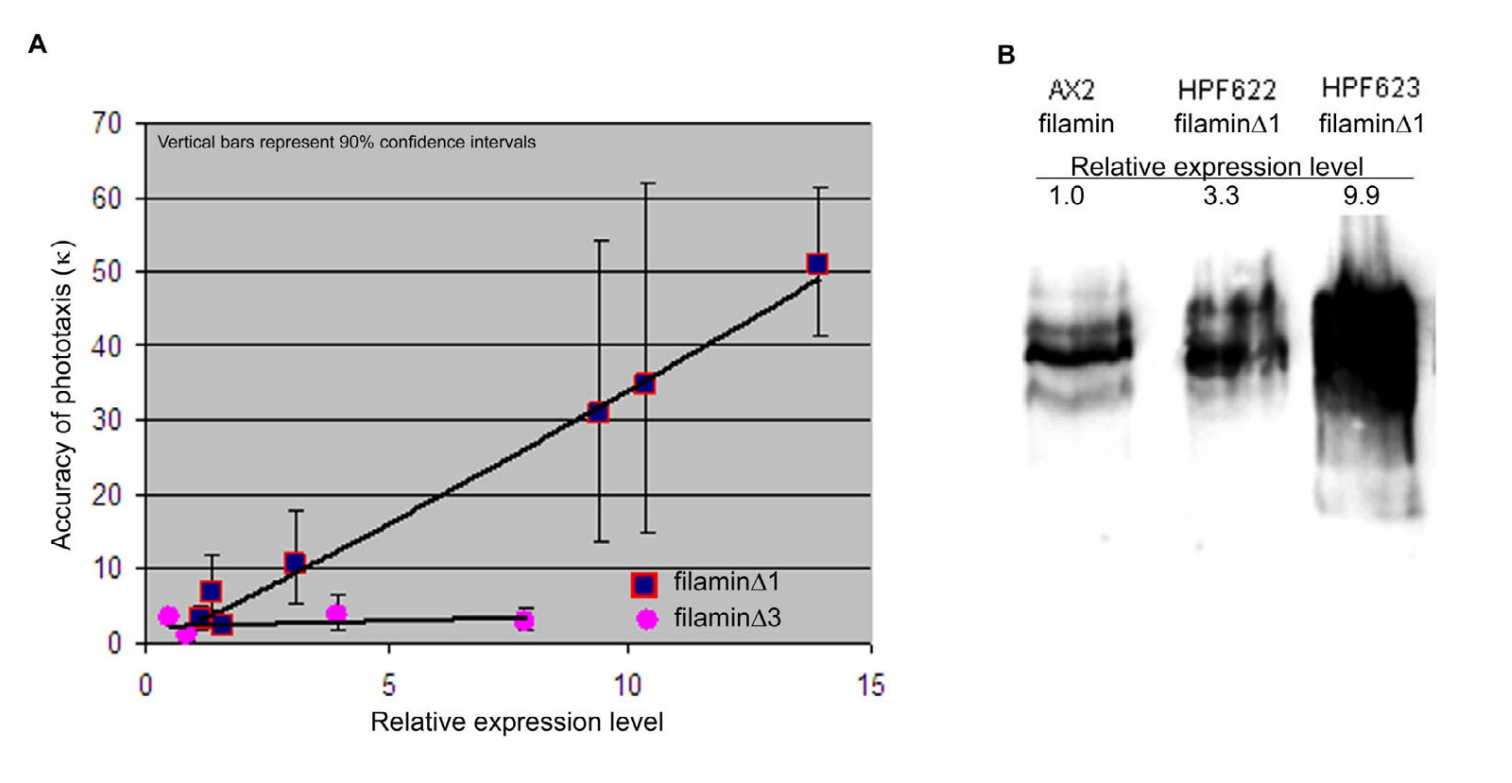
**Transformants expressing filaminΔ1 show increased accuracy of phototaxis in response to increased expression levels**. (A) The expression level of mutant filamin protein in several transformants was quantitated and normalised against wild type levels. As the level of filaminΔ1 expression increased, so too did the accuracy of phototaxis. Transformants expressing any of the other mutant filamin protein (filaminΔ3 used as an example in this figure) did not show this correlation increased expression had no effect on the accuracy of phototaxis. Vertical bars represent 90% confidence limits. (B) Western blot detecting filamin in wild type (AX2), and filaminΔ1 in two transformants HPF622 and HPF623. The amount of filamin protein detected was normalised against AX2 expression so that the level of filamin expressed in AX2 was 1. The level of filaminΔ1 expressed in HPF622 was 3.3 and these cells showed a relatively low accuracy of phototaxis whereas HPF623 cells expressed filaminΔ1 at 9.9 and showed a higher degree of accuracy of phototaxis.

To determine why the slug behavioural defects were rescued by filaminΔ1 in some strains but not others, a western analysis was performed to compare filamin expression levels in various transformants (Figure 6). The amount of filamin expressed from these transformants was measured relative to the expression of wild type filamin in AX2. It was found that as the level of filaminΔ1 expression increased so did the accuracy of phototaxis. Unlike filaminΔ1 transformants, those expressing any of the other mutant filamin proteins did not show increased accuracy of phototaxis with increased expression levels. This is illustrated in Figure 6 using transformants expressing filaminΔ3 as an example. Thus the difference in slug behavioural phenotypes amongst filaminΔ1-expressing transformants is due to differences in expression levels. The results suggest that, like wild type filamin, filaminΔ1 can support normal photo- and thermosensory signalling if expressed at high enough levels. However, the absence of repeat segment 1 appears to have reduced the efficiency with which this occurs, since effective complementation required hyperexpression of filaminΔ1 relative to normal wild type filamin levels. The results also confirmed that the repeat segments 2–6 are essential for normal slug phototaxis regardless of expression level.

### Localisation of filamin is unaffected by the absence of any of the repeat segments

**Figure 7:**
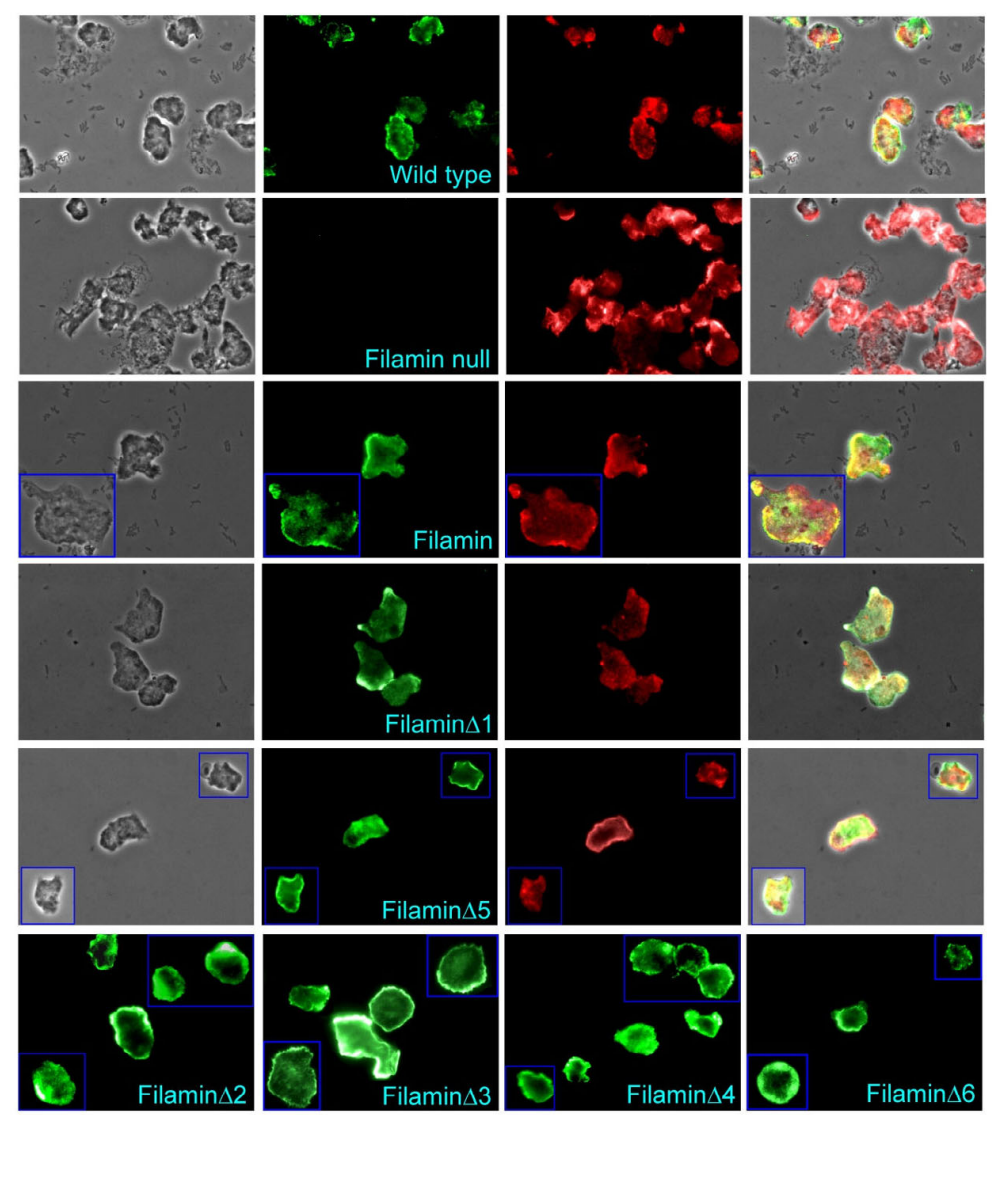
**Filamin and mutated filamin localise to the same actin rich areas**. Vegetative amoebae were viewed under a fluorescence microscope. For all but the last row, the first column contains phase contrast images, the second column contains the corresponding fields detecting green immunofluorescence using Alexa Fluor 488-conjugated anti-rabbit IgG and rabbit polyclonal antifilamin antibodies while the third column contains the corresponding field detecting actin stained with Texas Red-X phalloidin. The fourth column is an overlay of all three images and the yellow colour depicts regions where filamin and actin colocalise. Fluorescence images were obtained using an Olympus BX61TRF microscope under epifluorescence using blue light for excitation and either green or red emission filters. Images were obtained in grey scale and the colour balance adjusted *post hoc *to leave only the red or green channel as appropriate. As seen in column 2 filamin is found throughout the cells but is concentrated at the cell cortex. No filamin is detected in the filamin mutant HG1264. Strains expressing filaminΔ1 (which showed partial complementation of the phototaxis phenotype) and filaminΔ5 (which did not) were used as examples, as all strains expressing mutant filamins behaved in the same fashion. Column 3 illustrates that actin was found throughout the cell and was concentrated at the cell cortex. Comparison of columns 2 and 3 and examination of column 4 shows that in all strains tested filamin colocalises predominantly with actin at the cell cortex. The last row contains images of cells from the other strains tested expressing the various mutant filamins and depicts the localisation of filamin by immunofluorescence. As in all the other strains mutant filamin is concentrated at the cell cortex.

Full length filamin localises preferentially to regions of the cell that are rich in filamentous actin concentrating in the cell cortex, pseudopodia and lammelipodia [26]. It is known that the actin binding domain alone is sufficient for binding F-actin however it has been suggested that the rod domain regulates which actin rich structures filamin binds to with the full length filamin binding preferentially to the cortex and pseudopodia and the actin binding domain alone binding to all F-actin rich structures [27]. To determine whether any of the repeat segments are directly or indirectly involved in the localisation of filamin, the various transformants expressing either the full length filamin or one of the deletant filamins were analysed by fluorescence microscopy. Texas Red-X Phalloidin was used to detect actin and the antifilamin antibody in conjunction with an Alexa Fluor 488 conjugated secondary antibody was used to detect filamin. As seen in Figure 7, filamin colocalises with actin predominantly in the cell cortex and pseudopodia in all the strains tested, with the exception of HG1264 which showed no detectable filamin-associated fluorescence. These results suggest that none of the repeat segments are involved in the localisation of filamin, although we cannot exclude subtle, quantitative differences in the extent of filamin localization to the actin-rich cortex.

### The filamin-RasD interaction does not depend on any one repeat segment

**Figure 8:**
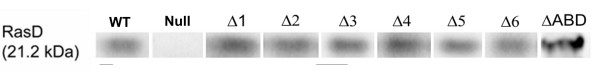
**RasD binding does not depend on any single repeat in the rod domain nor the actin binding domain**. Western blot using the antiRasD antibody to detect RasD in immunoprecipitations using protein extract from HPF616 (lane 1, WT) (AX2 ectopically expressing RasD), HPF631 (lane 2, Null) (HG1264 ectopically expressing RasD) and strains HPF632-638 (lanes 3–10) (HG1264 ectopically expressing RasD and one of filaminΔ1–6 or filaminΔABD as indicated). RasD is detected in all IP samples with the exception of the null mutant HPF631, indicating that filamin is required to pull down RasD and RasD interacts with filamin regardless of which portion of the scaffolding protein is absent.

Immunoprecipitation experiments previously described by Bandala-Sanchez *et al*. [18] showed that filamin and RasD interact together in a putative phototaxis signalling complex. Since RasD is required for phototaxis and thermotaxis, it was possible that the phototaxis defect in the filamin deletants was due to a failure to recruit RasD to the signalling complex. To determine whether individual repeats in the rod domain are required for the RasD-filamin interaction, immunoprecipitation experiments were performed using protein from strains ectopically expressing RasD in the absence of filamin or in conjunction with full length or deletant filamins. As seen in Figure 8, RasD immunoprecipitation was completely dependent upon the expression of filamin, but was unaffected by the lack of the actin binding domain or any one of the repeat segments – each internally deleted filamin protein was as effective in pulling down RasD as the full length protein. We conclude that the behavioural defects in these strains are not due to the inability of the deletant filamins to recruit RasD to the photosensory signalling complex.

## Discussion

In this study we have been able, by transformation with filamin expression constructs, to rescue the phototactic and thermotactic defects in the filamin null mutant HG1264. In a strain (HPF620) that expressed filamin to wild type levels phototaxis and thermotaxis were both completely restored to normal. The characterisation of strains expressing filaminΔ2–6 showed that repeat segments 2–6 are essential for filamins role in phototaxis and thermotaxis – filamin lacking any one of these repeat segments could not restore normal behaviour. By contrast, transformants expressing filamin with a deletion of the first repeat segment showed varying phototaxis phenotypes – some showed no restoration and others showed varying degrees of partial restoration of the phototaxis phenotype. The transformants were further analysed and it was revealed that the difference in degree of complementation of the phototaxis phenotype was directly related to the level of expression of filaminΔ1.

Previous work in this laboratory has suggested that Ddfilamin may act as a scaffold in a photosensory signalling pathway. Filamin's role as a scaffold would be to anchor and connect the various proteins involved in the photosensory pathway to the dynamic regulation of the actin cytoskeleton. It is known that human filamin, ABP280 can interact with a number of proteins and, with the exception of actin, all of these interactions required repeats in the rod domain. ABP280 contains 24 repeat segments and most of the interactions occur with repeats 14–24. Only one protein, PKC has been suggested to have a putative binding site somewhere between repeats 3–11 [4]. It seems then that most protein interactions preferentially occur with the C-terminal repeats. By analogy with human filamin, the first repeat in Ddfilamin may not be involved in any protein interactions, so that even in its absence all of the proteins involved in the complex can be assembled.

The deletions we introduced into each repeat removed the entire repeat except for a spacer consisting of the first two short β strands in the original repeat. As confirmed by the structural prediction in Figure 2, these deletions may maintain a similar distance and spatial relationship between the preceding and following repeat as would occur if the deleted segment was still present. However we cannot be certain that this is the case – the spacers may be unable to maintain the correct spatial relationships between preceding and following regions so that correct assembly of the photosensory signalling complex may be disturbed. In the case of the first repeat for example, the preceding region of the protein is the actin-binding domain and the spacer may change the angle, the extension or the flexibility of the protein between it and repeat segment 2. This may reduce the efficiency with which essential signalling proteins are recruited to other segments in the rod domain. Electron microscopy may be able to determine if the first two β strands that remained from each deleted repeat were still able to provide sufficient spacing between repeats and sufficient structural integrity.

The complementation achieved by filaminΔ1 overexpression suggests that, compared to wild type filamin, a smaller fraction of the mutant molecules can form functional filamin complexes and thus restore normal photo- and thermosensory signal transduction. This could result either from a reduced affinity of filaminΔ1 for one or more of its essential binding partners or from there being a functional and a nonfunctional fraction in the population of filamin molecules. This is not the case with the filamin protein lacking any of the other repeat segments, all of which seem to be absolutely required for phototaxis. This result is consistent with the findings of Khaire *et al. *[28] who, during the preparation of this manuscript, reported that phototaxis could not be restored by GFP-tagged deletant filamins lacking segments 2, 4 or 6 in their entirety (i.e. no spacer). By analogy with human filamin, these segments are more likely to be involved in directly binding heterologous signalling proteins so that regardless of how much mutant filamin is expressed some proteins would be missing from the complex and photosensory signalling would be defective.

In the case of other scaffolding proteins, it is known that their stoichiometry with respect to additional components in the signalling complex is tightly regulated to ensure sufficient concentrations of all components in the pathway are present. This was made evident through overexpression studies with several scaffolding proteins, an example of which is Ksr (Kinase suppressor of Ras). When Ksr is expressed at wild type levels it can function as a positive effector of Ras signalling whereas when it is overexpressed it inhibits Ras signalling [29]. This was suggested to result from the assembly of incomplete complexes because of competition for binding partners amongst the excess Ksr molecules. Our results suggest that in the filamin case, the supply of the scaffolding protein itself is the limiting factor in assembly of the signalling complexes for phototaxis. The full length filamin had to be expressed at least at wild type levels, and filaminΔ1 at higher than wild type levels to support normal or near normal photo- and thermosensory signalling.

In wild type cells filamin is present in abundance. It seems likely that for its availability to limit the formation of photosensory signalling complexes, Ddfilamin must participate in other competing processes that make only a fraction of it available for photoresponses. Mammalian filamin is known to play a role in various pathways such as regulating the internalisation of furin [30], the correct localisation of endosomes and lysosomes [30] and the ability to produce a stiffening response in response to shear stress [31]. Mutational studies have demonstrated that Ddfilamin is also likely to be involved in multiple pathways. These include incorporation of F-actin into the cytoskeleton after stimulation with chemoattractant, during pseudopod extension and in the process of phagocytosis [26,32,33]. Ddfilamin, with its potential to bind numerous proteins at all stages of development, may play a key role in the assembly of a variety of different signalling complexes each containing a specific set of proteins controlling specific cellular activities. Key proteins in these complexes may compete with one another for the available filamin. Such competitive binding has been described for the JIP4 scaffolding protein which is capable of binding both JNK and p38, but can only activate p38 when JNK is no longer bound [34].

It has been shown by Pang *et al. *[27] that the actin binding domain alone is sufficient for filamin binding to F-actin – immunofluorescence microscopy showed that all of the truncated filamin localised with actin mainly to the cell cortex and also to pseudopods. We found in this work that the subcellular localisation of filamin was not noticeably altered by the absence of any of the repeat segments. It has been suggested that the actin binding domain (ABD) alone does not exhibit the same spatial distribution as the full length filamin protein [27]. In particular it was suggested that the ABD localises to all actin rich areas whereas the full length filamin protein localises predominantly to newly formed pseudopods. However we observed no differences amongst the wild type or transformed strains expressing wild type filamin or strains expressing filamin with a deleted repeat segment. This suggests that no single repeat is responsible for regulating the localisation of filamin to specific actin structures. Khaire *et al. *[28] suggested on the basis of confocal laser fluorescence microscopy measurements that repeat segments 2 and 4 played subtle roles in the localisation of filamin, with mutant filamin protein still concentrating in the cortex, but 4 fold less strongly than the wild type filamin. Our visual examination of cells expressing mutant filaminΔ1–6 and full length filamin did not reveal any differences amongst the strains. However the fluorescence was not quantitated in our experiments and therefore subtle differences may not have been detected. Since Khaire *et al*. [28] did not measure expression levels, it is also possible that the increased levels of filamin they observed in the bulk cytoplasm compared to the cortex were a result of hyperexpression of the deletant filamin.

RasD has been shown to interact with filamin in a putative photosensory signalling complex [18]. Since filamin contains no recognizable Ras Binding Domains (RBD) (*i.e. *filamin is not a substrate for Ras activation), this interaction could be mediated indirectly through proteins bound to actin and thus to filamin through its actin-binding domain. More probably, RasD binding could be mediated directly or indirectly by one or more of the Ig-folded repeat segments. In an attempt to identify which repeat segment facilitates the filamin-RasD interaction, immunoprecipitations were performed using cells constitutively coexpressing RasD and filamin or RasD and one of each of the filamin deletants. These experiments showed that neither a single repeat segment nor the actin binding domain was solely responsible for the interaction. RasD may interact with filamin via more than one repeat segment so that when one is missing, RasD is still pulled down by the other repeat(s) to which it is bound. Although Ras proteins have been shown to bind directly to scaffolding proteins such as Raf-1, which contains a RBD, other scaffolding proteins have been shown to interact with Ras indirectly through additional proteins. An example of this is the MAPK cascade in which Ras interacts with the scaffolding protein Ksr through binding to Raf-1 which in turn interacts with Ksr indirectly via MEK [35]. This may also occur for the filamin-RasD interaction. The creation of filamin proteins lacking various combinations of multiple repeat segments may allow the determination of which segments are responsible for the RasD-filamin interaction.

## Conclusion

To support normal photosensory signalling, filamin must be present at wild type levels. Each of the repeat segments 2–6 is essential for filamins role in slug behaviour, however repeat segment 1 does not appear to be essential as hyperexpression of filaminΔ1 largely restored the slug behavioural defects. The repeats do not play a major role in the localisation of filamin to the actin rich cell cortex or the pseudopods. The recruitment of RasD to filamin complexes does not rely upon a single repeat segment in the rod domain or upon the actin binding domain and as such may interact indirectly through multiple repeat segments.

## Methods

### Dictyostelium strains and culture conditions

*Dictyostelium discoideum *wild type strain AX2, the filamin null mutant HG1264 [32] and the derived transformants were either grown axenically in HL-5 medium [36], which for transformed cell lines, contained 15 μg/ml G418 (Promega, Annandale, Australia) in shaken suspension (150 rpm) at 21°C or on SM agar plates with *Klebsiella aerogenes *as a food source [37]. The transformants used included HPF616 (transformant of AX2 ectopically expressing RasD under the control of the actin-15 promoter), HPF620 (HG1264 transformant expressing the full length filamin protein under the control of the actin-6 promoter), HPF629 and HPF639 (HG1264 expressing filamin under the control of the actin-15 promoter), HPF622, 623 and 637 (HG1264 transformants expressing the filamin protein with a deletion of repeat 1 of the rod domain under the control of the actin-6 promoter), HPF624-628 (HG1264 transformants expressing the filamin protein with a deletion of repeat 2–6 of the rod domain respectively, under the control of the actin-6 promoter) HPF631 (HG1264 transformant ectopically expressing RasD under the control of the actin-15 promoter) and HPF632-638 (HG1264 cotransformants ectopically expressing RasD under the control of the actin-15 promoter and filamin protein with a deletion of repeat 1–6 of the rod domain and deletion of the actin binding domain respectively).

### Plasmid Construction

The full length filamin gene (DDB0201554) was amplified using genomic or copy AX2 DNA as template and primers AWF (GCGGCGAGCTCCTCGAGTTAATTGGCAGTACGAGTAGTAG) and AWR (GCGGCAAGCTTGACATCTCTCTAACACCTGG).

The PCR product was cloned into the *Bam*HI and *Sac*I sites of pZErO™-2 (Invitrogen, Castle Hill, Australia) to create the construct pPROF267. In order to create deletions of a segment of each repeat in the rod domain long inverse PCR was employed using the Long Template PCR Kit (Roche, Castle Hill, Australia). The construct pPROF267 was used as template along with the following primers to delete segments of repeats one through to six: DEL1R (CGCTCTAGAGCTGACGGCGGATTTATTAACGAAACC), DEL1F (CGCTCTAGAGTCGACGACGGTTCAGATGCTCAACAC), DEL2R (CGCTCTAGAGTCGACTGGAACACCAACTTTACCACC), DEL2F (CGCTCTAGAGTCGACCTCAACTCTGACTCTCAAAAC), DEL3R (CGCTCTAGAGTCGACAGTTGGACGTTTAGCTTGGGC), DEL3F (CGCTCTAGAGTCGACGCTGATCCAGAGAAATCATAC), DEL4R (CGCTCTAGAGTCGACGCTTGGTTGGAAACATTCACC), DEL4F (CGCTCTAGAGTCGACCCAGCCCCATCCGCTGAACAC), DEL5R (CGCTCTAGAGTCGACGAATTCAGCTGGGGCATTATC), DEL5F (CGCTCTAGAGTCGACGTCAAATGCATTGAAGGTGCC), DEL6R (CGCTCTAGAGTCGACAGTGAATGAACCGAATGATGA), DEL6F (CGCTCTAGAGTCGACCTTGGTAACCCAGGTAAAAAG). RODF (CGCTCTAGAGTCGACGCCAGCAAGGTTGAAGTTTATGGT), RODR (CGCTCTAGAGTCGACTCCACTTGGAGCAGCAGCCAT).

These long PCR products were digested with *Sal*I and religated to form constructs containing the pZErO™-2 backbone and the filamin gene with a deletion of repeat one through to six in the rod domain and deletion of the actin binding domain. These deletant filamin genes were subcloned as was the full length filamin gene into the *Dictyostelium *expression vector pDNeo2 [38] via the *Pst*I and *Sac*I sites. These constructs were named pPROF389 which contained the full length filamin gene, pPROF438-443 which contained deletions of repeat segments one through six in the rod domain respectively and pPROF445 which contained a deletion of the actin binding domain. The full length filamin gene was also subcloned from pPROF267 into the *Dictyostelium *expression vector pPROF257 via the *Not*I and *Sac*I sites. This construct was named pPROF354.

### Antibody Production

Polyclonal antibodies directed against RasD and the rod domain of filamin were produced by the Institute of Medical and Veterinary Science (IMVS, Adelaide, Australia) as described by Bandala-Sanchez *et al. *[18].

### Protein isolation and Western Analysis

Cells were harvested from axenically growing cultures via centrifugation in an Eppendorf centrifuge (Model 5702) at 1,900 × *g *for 2 min. The pelleted cells were washed three times in saline solution, resuspended in 1 ml Lysis Buffer per 10^7 ^cells (50 mM Tris-HCl, 120 mM NaCl, 1% NP-40 and protease inhibitors (Roche)) and incubated on ice for 20 min. The lysate was then centrifuged at 10,000 × g for 20 min in a benchtop Eppendorf centrifuge at 4°C to pellet the cell debris and the supernatant was transferred to a new tube and stored at -70°C. The concentration of the protein samples was determined using the Bradford method and the Bio-Rad Protein Assay with BSA as a standard. Laemmli buffer was added to the protein samples which were boiled for 5 min and subjected to SDS-PAGE on 8 or 12% gels. The separated proteins were transferred to PVDF membranes (Amersham, Arlington Heights, IL, USA) and antiRasD or antifilamin antibodies were used to detect RasD and filamin respectively. Membranes were blocked with TBS (10 mM Tris-HCl, 150 mM NaCl, pH 7.5) containing 5% skim milk powder for at least 1 hr at room temperature. The membranes were briefly washed in TBST (20 mM Tris-HCl, pH 7.5, 500 mM NaCl, 0.05% Tween20) and incubated with primary antibody (1:500) in TBS containing 5% skim milk powder overnight at 4°C. The membrane was washed three times with TBST and incubated with anti-rabbit HRP conjugated secondary antibody (Promega, Madison, WI, USA) 1:2,500 for 1 hr at 4°C followed by three washes in TBST. The signals were detected using the enhanced chemiluminescence system (ECL, Amersham).

### Immunoprecipitations

All steps were performed at 4°C to prevent degradation of protein. Five microliters of antifilamin antibody were added to 300 μl of protein extract and incubated shaking overnight prior to incubation with 20 μl of protein A-sepharose for 2 hrs. The immunoprecipitate was centrifuged at 9,000 × g for 5 min and the supernatant was discarded. The protein A-sepharose was washed four times in NETN buffer (20 mM Tris-HCl, pH 8.0, 1 mM EDTA, 0.5% NP-40, 900 mM NaCl) and once in NETN buffer without NaCl. The pelleted beads were resuspended in 50 μl Laemmli buffer.

### Immunofluorescence Microscopy

*D. discoideum *amoebae were grown in HL-5 medium on sterile coverslips in six-well Costar plate wells (Nunc™) and equilibrated in Lo-Flo HL-5 medium [30] for 1 hr. Prior to fixation, the coverslips were washed twice in phosphate buffer (12 mM Na_2_HPO_4_, 12 mM NaH_2_PO_4 _[pH 6.5]) and the cells were flattened and fixed by placing the coverslips on a layer of 1% agarose in phosphate buffer containing 3.7% paraformaldehyde for 30 min. After fixation the cells were washed four times in PBS and cells were permeabilised with 100% methanol prechilled at -20°C. One unit of Texas Red-X phalloidin (Molecular Probes) was added to each coverslip and incubated for 30 min followed by three washes in PBS. The cells were blocked in blocking buffer (1% bovine serum albumin (type V, Sigma), 1% cold-water fish-skin gelatin (Sigma), 0.05% Tween 20 in PBS) for 1 hr, and then incubated with antifilamin antibody diluted 1/100 in blocking buffer overnight at 4°C. Cells were washed three times in PBS containing 0.05% Tween 20 and incubated with secondary antibody (goat anti-rabbit Alexa-Fluor 488 (Molecular Probes™ Invitrogen™) diluted 1/500 in blocking buffer for 45 min at room temperature. The coverslips were washed four times in PBS containing 0.05% Tween 20, air dried and mounted on a microscope slide with 90% glycerol in PBS. Cells were observed on an Olympus BX61TRF microscope and digital images were captured using an Olympus U-CMAD3 camera.

### Phototaxis and Thermotaxis Assays

Qualitative phototaxis tests were performed as described in Darcy *et al. *[6] by transferring a toothpick scraping of amoebae from a colony growing on a *K. aerogenes *lawn to the center of charcoal agar plates (5% activated charcoal, 1.0% agar). Phototaxis was scored after a 48 hr incubation at 21°C with a lateral light source. Quantitative phototaxis tests involved the harvesting of amoebae from mass plates, thoroughly washing them free of bacteria, suspending them in saline at the appropriate dilutions and inoculating 20 μl onto a 1 cm^2 ^area in the center of each charcoal agar plate. The resulting cell densities ranged from about 1.5 × 10^6 ^to 3.7 × 10^7 ^cells/cm^2^. The phototaxis was again scored after 48 hrs incubation at 21°C with a lateral light source. Quantitative thermotaxis utilized washed amoebae prepared as for quantitative phototaxis and plated at a density of 3 × 10^6 ^amoebae/cm^2^. A 20 μl aliquot of cells at this dilution were plated onto a 1 cm^2 ^area in the center of water agar plates (1.0% agar) and incubated for 72 hrs in darkness on a heat bar producing a 0.2°C/cm gradient at the agarose surface. The arbitrary temperature units correspond to the temperatures 14°C at T1 and increasing in 2°C increments to 28°C at T8, as measured at the center of plates in separate calibration experiments. Slug trails were transferred to PVC discs, stained with Coomassie Blue and digitized. The orientation of the slug migration was analysed using directional statistics [3].

## Authors' contributions

SJA created the antifilamin antibody and all the strains except HPF616, performed the phototaxis and thermotaxis assays, the analysis of strains, and drafted the manuscript. EBS created the antiRasD antibody and strain HPF616. AA performed the immunofluorescence microscopy work and PRF participated in the design and helped to draft the manuscript. All authors read and approved the final manuscript.
